# Draft genome sequence of the *Alicyclobacillus acidocaldarius* strain LH1, isolated from a highly acidic solfatara field

**DOI:** 10.1128/mra.00776-24

**Published:** 2025-06-18

**Authors:** Francisca J. Fuentes-Tobar, Nidia Torres-Ponce, Jenny M. Blamey, Sara Cuadros-Orellana

**Affiliations:** 1 Escuela de Ingeniería en Biotecnología (IBT), Universidad Católica del Maule 28048, Talca, Maule Region, Chile; 2 Doctorado en Biotecnología Traslacional (DBT), Universidad Católica del Maule 28048, Talca, Maule Region, Chile; 3 Fundación Científica y Cultural Biociencia, Santiago, Chile; 4 Departamento de Biología, Facultad de Química y Biología, Universidad de Santiago de Chile 682971, Santiago, Santiago Metropolitan Region, Chile; 5 Centro de Biotecnología de los Recursos Naturales, Universidad Católica del Maule 28048, Talca, Maule Region, Chile; California State University San Marcos, San Marcos, California, USA

**Keywords:** *Alicyclobacillus*, genome, antibiotic resistance

## Abstract

The *Alicyclobacillaceae* are known for their ability to produce thermostable industrial catalysts, including endonucleases and hydrolases. Here, we report the draft genome sequence of the *Alicyclobacillus acidocaldarius* strain LH1, isolated from a highly acidic Andean solfatara in the Chilean Andes.

## ANNOUNCEMENT

*Alicyclobacillus* sp. is a thermo-acidophilic, Gram-positive, aerobic, spore-forming bacteria (1) often found in soil (2), volcanic environments (3), mining sites (4), and acidic food and beverages (5).

Strain LH1 was isolated in September 2022 from the “Los Hoyos” solfatara field (–35.9525464 and –70.7058126). Samples from steaming soil at 67.6°C, pH 2.5, were enriched in DSMZ Medium 88 ([NH_4_]2SO_4_ [1.30 g/L], KH_2_PO_4_ [0.28 g/L], MgSO_4_ × 7 H_2_O [0.25 g/L], CaCl_2_ × 2 H_2_O [0.07 g/L], FeCl_3_ × 6 H_2_O [0.02 g/L], yeast extract [1.00 g/L], glucose [1.00 g/L], and 1 mL/L Wolfe vitamins solution, pH 3.0), 70°C, for 48 hours. Isolation was achieved through serial dilutions to extinction in the same medium.

Genomic DNA was obtained using a previously reported protocol (6) and randomly sheared into ~350 bp fragments. A whole-genome library was prepared using the NEBNext DNA Library Prep Kit, checked with an Agilent 2100 bioanalyzer, quantified using qPCR, and sequenced using an Illumina NovaSeq 6000 platform and a PE150 strategy at the Novogene Corporation Inc. (Sacramento, CA, USA).

A total of 4,873,788 raw reads were obtained, checked with FastQC v0.12.1, and trimmed with Trimmomatic v0.39 (7) using ILLUMINACLIP 2:30:10, SLIDINGWINDOW:4:20, and MINLEN:50. Surviving reads were assembled using Megahit v1.2.9 (8).

**Fig 1:**
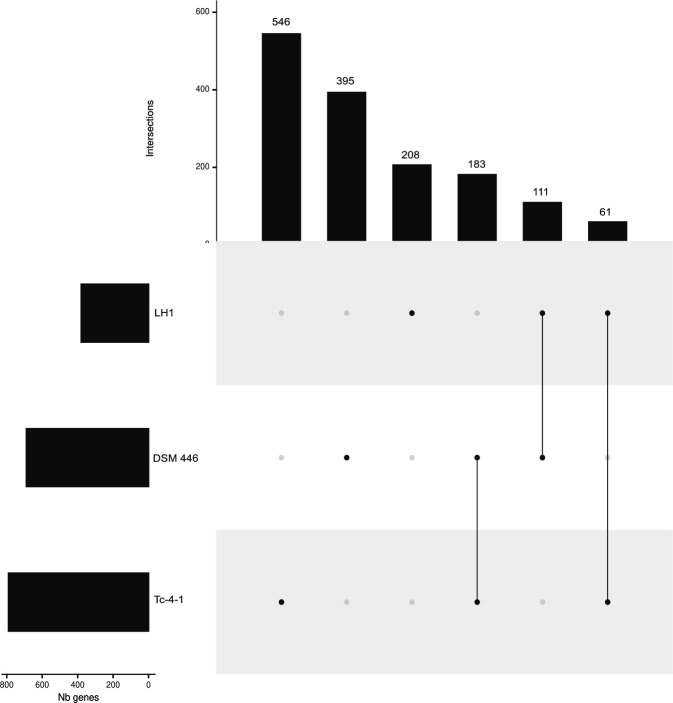
Upset diagram for accessory genes of strains DSM446, Tc-4–1, and LH1 of *A. acidocaldarius*. The total size of each set is represented on the left barplot. Every possible intersection is represented by the bottom plot, and their occurrence is shown on the top barplot. Each row corresponds to a possible intersection; the filled-in cells show which set is part of an intersection.

Assembly quality and completeness were assessed with QUAST v5.0.2 (9) and BUSCO v3.0.2 (10). The genome was annotated using the NCBI PGAP v1.2. pipeline, and strain identity was confirmed as *Alicyclobacillus acidocaldarius subsp. acidocaldarius* by the Similar Genome Finder service through BV-BRC (11) and FastANI v1.3 (12). CRISPRCasMeta (online) (13), mobileOG-db v1.1.3 (14), Phigaro v1.0.1 (15), and VirSorter v2.2.4 (16) were used to annotate antiviral systems, mobile genetic elements, and prophages. The draft genome is 2,602,585 bp long, comprising 170 contigs ranging from 1,008 bp to 112,740 bp, with an N50 of 33,932 bp, an L50 of 25, and a GC content of 63.5%. Genome completeness is 89.7%. The draft genome contains 2,674 genes, including 2,529 coding DNA sequences (CDSs), 60 tRNA genes, and 7 rRNA genes, including a complete 16S rRNA 99.35% similar to that of DSM446 (*A. acidocaldarius* type strain). The average nucleotide identity between strains LH1 and DSM446 (reference genome, GCF_000024285.1) is 97.21%, according to FastANI. Three CDS coding for endonucleases with biotechnological interest were found, namely: endonuclease III (EC 4.2.99.18), endonuclease IV (EC 3.1.21.2), and endonuclease Q. Furthermore, strain LH1 harbors 5 Cas systems and 43 mobile features, including 13 related to integration/excision, 16 related to replication/recombination/repair, and 10 involved in host-phage interaction. A pangenome analysis including different genomes of *A. acidocaldarius*, including strain LH1 (this work), was performed using Roary (17) implemented in PanExplorer (online) (18). Strain LH1 contributes 208 unique genes to the species pangenome, as compared to the 395 and 546 unique genes contributed by strains DSM446 and Tc-4–1, respectively (Fig. 1).

## Data Availability

The genome sequence of *Alicyclobacillus acidocaldarius* strain LH1 was deposited in GenBank with the accession number GCA_040779485.1 and raw reads with SRR30001008. The associated BioProject and BioSample accession numbers are PRJNA1128703 and SAMN42091777, respectively. Genome annotation made using CRISPRCasMeta (online), ResFinder (online), mobileOG-db v1.1.3., and VirSorter v2.2.4 is available in the Zenodo database under DOI 10.5281/zenodo.13994080.
